# ECM1 regulates the resistance of colorectal cancer to 5-FU treatment by modulating apoptotic cell death and epithelial-mesenchymal transition induction

**DOI:** 10.3389/fphar.2022.1005915

**Published:** 2022-11-02

**Authors:** Sirui Long, Jie Wang, Fanbin Weng, Zhigang Pei, Shixian Zhou, Guiyin Sun, Debing Xiang

**Affiliations:** ^1^ Department of Oncology, Chongqing University Jiangjin Hospital, Chongqing, China; ^2^ Department of Oncology, Jiangjin Central Hospital of Chongqing, Chongqing, China; ^3^ Department of Pathology, Chongqing University Jiangjin Hospital, Chongqing, China; ^4^ Department of Pathology, Jiangjin Central Hospital of Chongqing, Chongqing, China

**Keywords:** ECM1, drug resistance, colorectal cancer, cell apoptosis, epithelial-mesenchymal transition, PI3K/AKT/GSK3βsignaling pathway

## Abstract

5-Fluorouracil (5-FU) chemoresistance is a persistent impediment to the efficient treatment of many types of cancer, yet the molecular mechanisms underlying such resistance remain incompletely understood. Here we found CRC patients resistant to 5-FU treatment exhibited increased extracellular matrix protein 1 (ECM1) expression compared to CRC patients sensitive to this chemotherapeutic agent, and higher levels of ECM1 expression were correlated significantly with shorter overall survival and disease-free survival. 5-FU resistant HCT15 (HCT15/FU) cells expressed significantly higher levels of ECM1 relative to parental HCT15 cells. Changes in ECM1 expression altered the ability of both parental and HCT15/FU cells to tolerate the medication *in vitro* and *in vivo via* processes associated with apoptosis and EMT induction. From a mechanistic perspective, knocking down and overexpressing ECM1 in HCT15/FU and HCT15 cell lines inhibited and activated PI3K/AKT/GSK3β signaling, respectively. Accordingly, 5-FU-induced apoptotic activity and EMT phenotype changes were affected by treatment with PI3K/AKT agonists and inhibitors. Together, these data support a model wherein ECM1 regulates CRC resistance to 5-FU *via* PI3K/AKT/GSK3β pathway-mediated modulation of apoptotic resistance and EMT induction, highlighting ECM1 as a promising target for therapeutic intervention for efforts aimed at overcoming chemoresistance in CRC patients.

## Introduction

Colorectal cancer (CRC) is the third leading cause of mortality and accounts for about 9.4% of all types of cancer (Sung et al., 2021). This disease’s lethal character is primarily due to the high rates of chemoresistance and recurrence (Wu et al., 2022). 5-Fluorouracil (5-FU) remains a promising therapy option for CRC. However, treatment failure is often due to drug resistance, ultimately contributing to further tumor progression and patient death (Van der Jeught et al., 2018). 5-FU resistance of colorectal cancer is the main cause of CRC chemotherapy failure and leads to disease progression and death (Vodenkova et al., 2020). It may be contributed to evasion of apoptosis (de la Cruz-Morcillo et al., 2012; Blondy et al., 2020), changes in the cell cycle and DNA-damage repair machinery (Azwar et al., 2021), regulation of autophagy (Kocaturk et al., 2019), epithelial-to-mesenchymal transition (Escalante et al., 2021), etc. Nevertheless, the underlying molecular mechanisms are still unknown.

Extracellular matrix 1 (ECM1) was first identified as an 85 kDa glycoprotein secreted by a murine osteogenic stromal cell line MN7 (Mathieu et al., 1994). In recent studies, ECM1 has been shown to play an essential role in epithelial malignancies. The expression of ECM1 is much higher in a variety of tumor tissues, such as ovarian cancer (Yin et al., 2021), CRC (Long et al., 2022), breast cancer (Steinhaeuser et al., 2020), gastric cancer (Wu et al., 2014; Liu et al., 2019), etc., when compared with normal tissues. In our previous study, we found that high expression of ECM1 was correlated with tumor size, lymph node status and TNM stage in CRC patients and functionally promoted CRC cells proliferation, invasion and migration (Long et al., 2022). However, whether ECM1 also influence the procession of CRC resistance to 5-FU has not been clarified. So this study aims to explore the role of ECM1 in 5-FU resistance of CRC to provide a potential therapeutic target to overcome chemoresistance in CRC patients and prolong their survival.

## Materials and methods

### Clinical samples

Tissue samples were collected from 111 CRC patients receiving 5-FU treatment after radical surgery at Chongqing University Jiangjin Hospital between January 2016 and December 2020. The Chongqing University Jiangjin Hospital’s institutional ethics committee approved this study (Approval No. KY20200010), and all participants provided consent forms.

### Immunohistochemistry

Prepared tissue slices (3 μm thick) were stained with an IHC staining kit (ZSGB-BIO, China) according to the manufacturer’s instructions. Slides were treated overnight at 4°C with anti-ECM1 (1:250) (Abcam, United Kingdom), E-cadherin (1:100) (Affinity, China), N-cadherin (1:100) (Affinity, China), Bcl-2 (1:100) (Affinity, China), Bax (1:100) (Affinity, China), Caspase3 (1:100) (Affinity, China), washed, incubated with secondary antibodies for 30 min, and then analyzed. The percentage of stained cells was used for IHC scoring (1 = < 25%, 2 = 25%–50%, 3 = 51%–75%, and 4 = >75%), with these scores being multiplied by staining intensity scores (0, 1, 2, or 3) to yield final IHC scores ranging from 0 to 12. A score = < 4 corresponded to low ECM1 expression levels, whereas scores >4 indicated high ECM1 expression.

### Cell culture

HCT15, HCT15/FU, and 293T cells bought from the BeNa Culture Collection (BeNa, China) were utilized for this study. While 293T-cells were cultivated in DMEM (Gibco) for 293T-cells, HCT15 and HCT15/FU were grown in RPMI-1640. Cells were treated with 30 µM 740 Y-P or 50 µM LY294004 for 24 h respectively in different groups. Penicillin/streptomycin and 10% FBS were added to the media, and all cells were cultured at 37°C in an incubator with a humidified 5% CO_2_ atmosphere.

### Lentiviral transduction

The appropriate psPAX2 and pMD2. G lentiviral packaging plasmids were used to transfect 293T-cells with the pLVX-CMV-IRES-EGFP-puro empty vector, PSIH1-H1-copGFP-T2A-PURO empty vector, PSIH1-H1-copGFP-T2A-PURO-shECM1 vector, or pLVX-CMV-IRES. Cell supernatants were collected after 72 h of incubation, centrifuged at 3,000 rpm for 20 min, filtered, concentrated, then centrifuged again for 20 min at 5,000 rpm while kept at 4°C. After infecting cells with these lentiviral preparations, stably transduced cells were selected using puromycin dihydrochloride.

### qRT-PCR

Trizol (Invitrogen, United States) was used to extract cellular RNA, and the ReverTra Ace^®^ qPCR RT Kit was used to synthesize cDNA (Toyobo, Japan). The Applied Biosystems 7300Plus machine (Applied Biosystems, United States) and SYBR^®^ Green Realtime PCR Master Mix (Toyobo, Japan) were used for all qRT-PCR analyses. Relative gene expression was examined *via* the 2^−ΔΔCT^ approach. Primers were as follows: β-actin-R: 5′-TCC​CGG​CCA​GCC​AGG​TCC​A-3′; β-actin-F: 5′-ACC​CCG​TGC​TGC​ACC​GAG-3′, ECM1-R:5′-TTGGCGTTCTCAGTGTCTCC-3’; ECM1-F: 5′-CCT​GGG​CTG​ATC​CAC​AAC​AT-3′.

### Western immunoblotting

Protein lysates were prepared, and equal amounts of samples (20–30 µg for cell samples, 50–60 µg for tissue samples) were separated *via* 8%, 10%, or 12% SDS-PAGE based on target protein molecular weight values. PVDF membranes (Millipore, United States) were used to transfer the proteins, which were subsequently blocked with nonfat milk at 5% for 2 h. The blots were then probed overnight at 4°C with primary antibodies diluted in TBST. After three washes, blots were probed for 2 h with secondary antibodies (ZSBIO, China), and proteins were identified using a chemiluminescence imaging system (Tanon, China). Utilized antibodies were specific for E-cadherin (CST, United States), p-PI3K (Abcam, United Kingdom), PI3K (Abcam, United Kingdom), p-AKT (Abcam, United Kingdom), AKT (Abcam, United Kingdom), ECM1 (Abcam, United Kingdom), N-cadherin (CST, United States), p-GSK3β (Novus, United States), HRP-labeled Goat Anti-Rabbit IgG (ZSGBIO, China), GSK3β (Novus, United States), Bcl-2 (Affinity, China), Bax (Affinity, China), Caspase3 (Affinity, China) and GAPDH (CST, United States), and all were diluted 1:800–1:1000.

### Drug sensitivity assays

After being seeded in 96-well plates with 7000 cells per well for 24 h, the cells were incubated for 72 h in 100 µl of RPMI-1640 containing 5-FU doses. Then, 10 µl of CCK-8 reagent and 90 µl of RPMI-1640 were added to each well (Tongren Company, Japan). OD_450_ was discovered using a microplate reader (BMG LABTECH, German) after 2 h of incubation at 37°C.

### Colony formation assays

After 48 h, 1000 cells in 6-well plates were treated with the appropriate concentrations of DMSO or 5-FU. Cells were stained for 3 min at room temperature when cellular colonies were visible with 0.2% crystal violet after being fixed for 30 min with 4% paraformaldehyde (PFA).

### Wound healing assays

In 6-well plates, cells were planted at a density of 1 × 10^6^ per well. The cell monolayer was scraped with a 200 µl pipette tip when it reached 90% confluence. Following a PBS rinse, cells were cultured for 48 h in a medium supplemented with 1% FBS and a range of 5-FU concentrations. Wound healing rates were calculated by comparing the area between wound edges using ImageJ as follows: Wound healing rate (%) = (area at 0 h—area at 48 h)/area at 0 h × 100 %.

### Transwell assays

24-well Transwell inserts (Corning, United States) with 8 μm holes were used for all cellular migration and invasion studies, either uncoated or coated with a 9:1 combination of RPMI-1640 and Matrigel (Corning, United States). Cells were treated with appropriate 5-FU concentrations, after which they were added to the upper chamber of these inserts (2 × 10^5^ cells in 200 µl), with 600 μl of media containing 20% FBS added to the lower chamber. Cells were fixed with 4% PFA for 30 min after being stained with crystal violet for 3 min after 24 h, and non-invasive/non-migratory cells were removed using cotton swabs.

### Apoptotic staining assays

A One-Step TUNEL Apoptosis Assay Kit (Beyotime, China) was utilized based on provided directions to detect apoptotic CRC cells. In separate experiments, a flow cytometry-based approach was used to detect apoptosis by culturing cells in 6-well plates (3 × 10^5^/well) for 24 h, after which the cells were treated for 48 h with appropriate 5-FU concentrations. After that, the cells were collected, washed three times with PBS, and stained with an Annexin V-FITC Apoptosis Detection Kit (Beyotime, China). Flow cytometry (BD, United States) was then used to detect apoptotic death rates within 1 h.

### Animal model experiments

Male BALB/c mice that were 4 weeks old were purchased from the Shanghai Laboratory Animal Center and kept there with free access to food and water on a regular day/night cycle. The university’s Institutional Ethics Committee authorized all animal experiments conducted at Chongqing University after their procedures were reviewed and found to comply with the Guide for the Care and Use of Laboratory Animals. Subcutaneous implantation of a suspension of HCT15/FU-NC or HCT15/FU-shECM1 cells (2×10^6^ cells/50 µl) into animals in the right flank created a xenograft mouse model system. At 1 week post-inoculation, animals were separated into the following 4 groups (*n* = 3/group): HCT15/FU-NC (DMSO), HCT15/FU-NC (5-FU), HCT15/FU-shECM1 (DMSO), and HCT15/FU-shECM1 (5-FU). Every 7 days, DMSO or 5-FU (25 mg/kg) was delivered intraperitoneally (i.p.) to the relevant mice groups. After 4 weeks of treatment, mice were put to death, and tumors were collected, weighed, and utilized in further studies. The 5-FU-mediated tumor inhibition rate was calculated as follows (Li et al., 2022): Tumor inhibition rate = V1-V0/V0*100, where V1 and V0 respectively refer to the tumor volume at the end and beginning of treatment.

### Statistics analysis

GraphPad Prism 9.0 (GraphPad Software, California), ImageJ (National Institutes of Health, United States), and SPSS 26.0 (International Business Machines Corporation, United States) were used to analyze the data. Data were compared using two-tailed t-tests and presented as means ± standard deviation (x ± s). All *in vitro* tests were conducted three times. *p < 0.05, **p < 0.01, ***p < 0.001, and ****p < 0.0001.

## Results

### Extracellular matrix 1 is associated with 5-Fluorouracil resistance and poor prognosis in colorectal cancer patients

**FIGURE 1:**
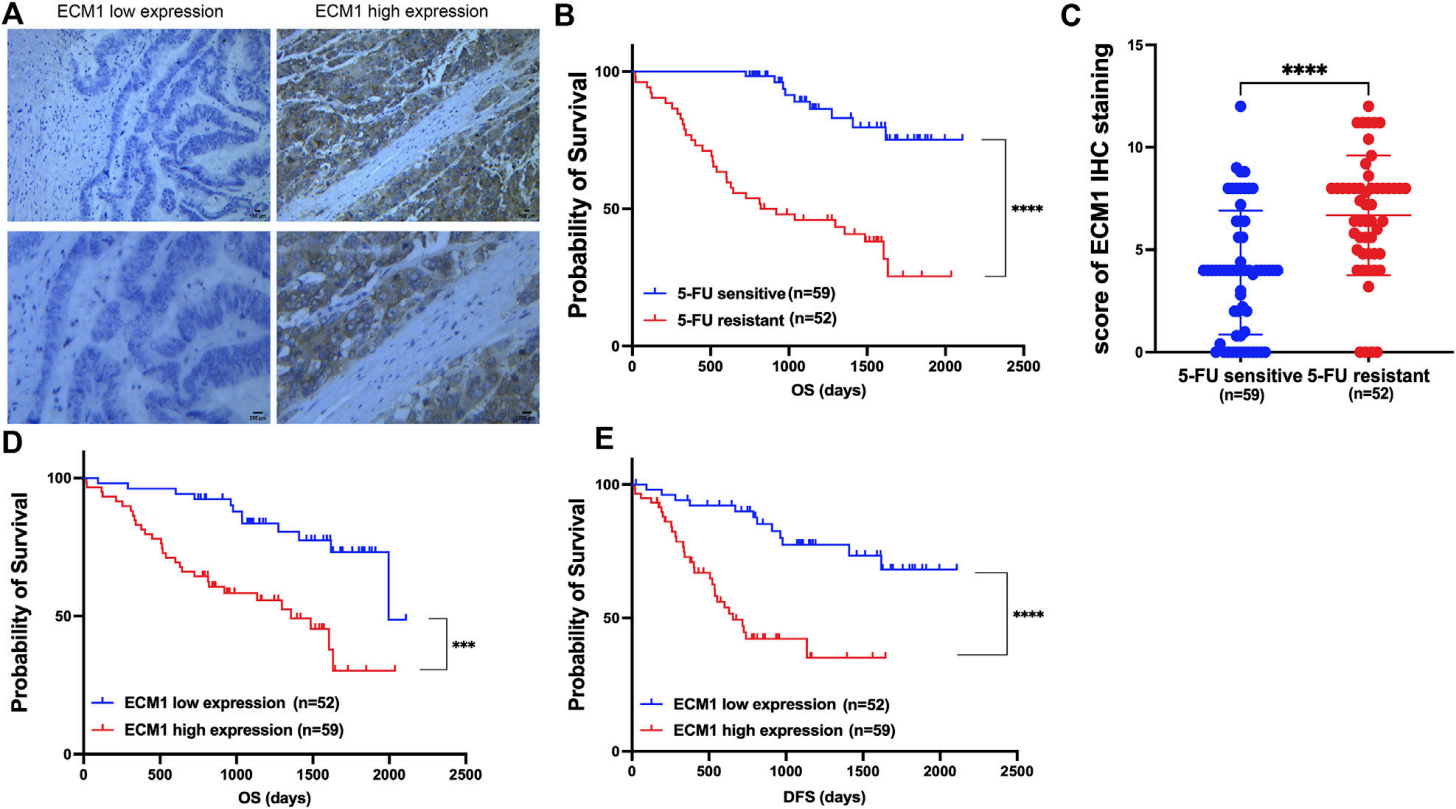
ECM1 is associated with 5-FU resistance and poor prognosis in CRC patients. **(A)** Photographs illustrating the presence of ECM1 protein in tumor tissue samples from CRC patients. **(B)** Kaplan-Meier analyses of the OS of 5-FU-sensitive and -resistant patients. **(C)** The ECM1 expression of 5-FU-resistant and -sensitive patient samples was assessed. **(D,E)** The OS **(D)** and DFS **(E)** of patients expressing low and high levels of ECM1 were compared. Data are means ± SD (***p < 0.001, ****p < 0.0001).

Following surgery, samples from CRC patients receiving 5-FU-based drug regimens, such as capecitabine, were taken to test the hypothesis that ECM1 could control 5-FU resistance in CRC. ECM1 levels in these samples were identified using IHC (Figure 1A). A previous report (Fang et al., 2022) defined 5-FU resistance based on patients with disease-free survival (DFS) of <2 years, whereas patients with a DFS ≥2 years were considered sensitive to 5-FU. Based on these criteria, 5-FU-resistant patients exhibited decreased overall survival (OS) relative to 5-FU-sensitive patients (Figure 1B), and ECM1 expression levels were significantly higher among 5-FU-resistant patients as compared to sensitive patients (Figure 1C). Notably, higher ECM1 expression levels were related with lower OS and DFS when compared to lower ECM1 expression levels (Figures 1D,E).

### Extracellular matrix 1 is involved in 5-Fluorouracil resistance in colorectal cancer cells

**FIGURE 2:**
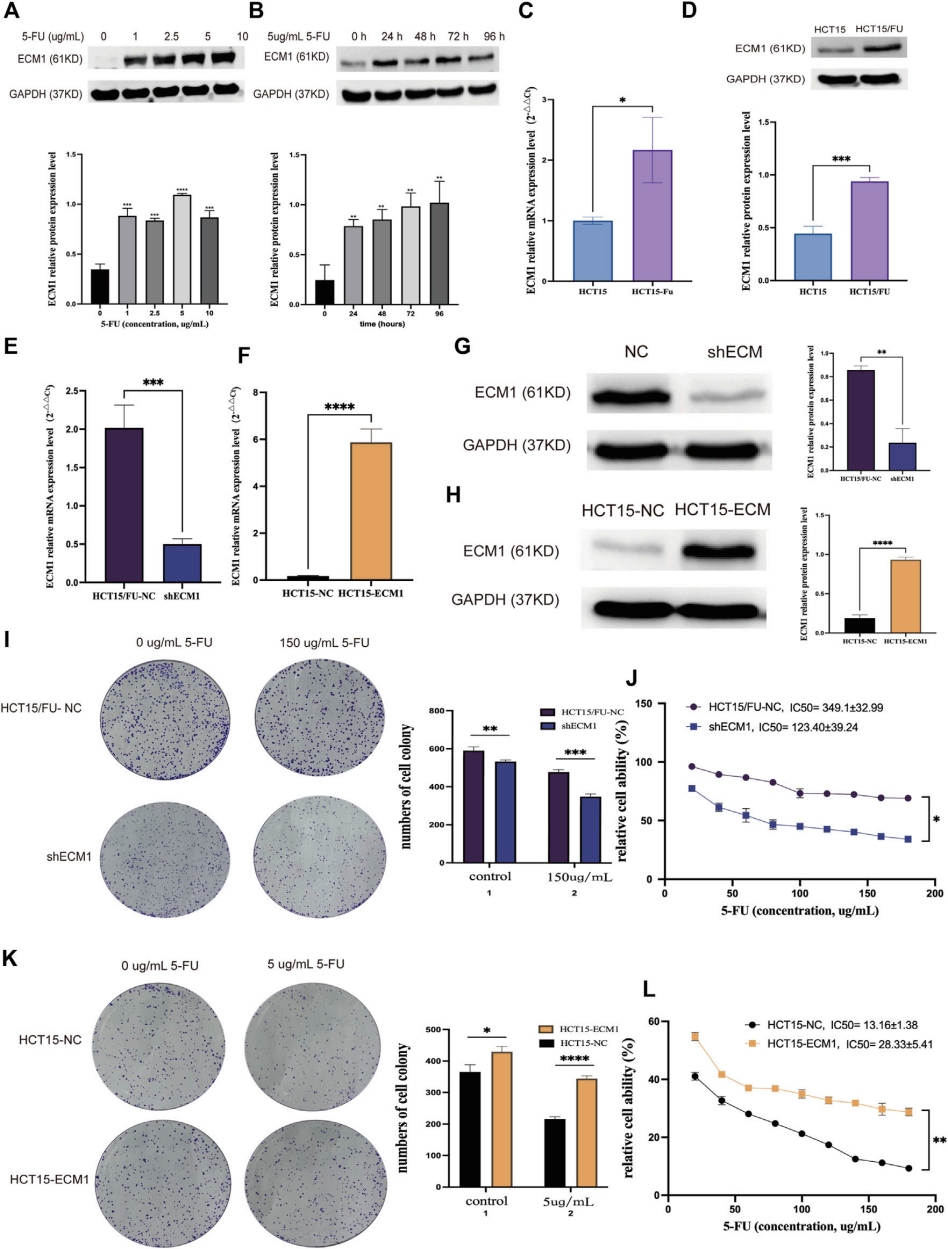
ECM1 is involved in 5-FU resistance in CRC cells. **(A)** After treatment for 24 h with a range of 5-FU doses (0, 1, 2.5, 5, or 10 µg/ml), ECM1 levels in HCT15 cells were detected *via* Western immunoblotting. **(B)** After treatment for 24, 48, 72, or 96 h with 5 µg/ml of 5-FU, ECM1 protein levels in HCT15 cells were detected *via* Western immunoblotting. **(C,D)** qPCR and Western immunoblotting approaches detected ECM1 levels within HCT15 and HCT15/FU cells. **(E–H)** Western immunoblotting and qPCR were used to evaluate the efficacy of ECM1 overexpression or knockdown. Through CCK-8 and colony formation tests, the susceptibility of these cells to 5-FU was further examined. **(I,J)** The effects of ECM1 knockdown on 5-FU resistance in HCT15/FU cells. **(K,L)** The effect of excessive ECM1 expression on 5-FU sensitivity of HCT15 cells. Data are means ± SD (*p < 0.05, **p < 0.01, ***p < 0.001, ****p < 0.0001).

Western immunoblotting was performed to analyze the expression of ECM1 in HCT15 cells after 24 h of treatment with a variety of 5-FU doses first to understand the functional relationship between ECM1 and the chemoresistance of CRC cells to 5-FU. This approach revealed that 5-FU induced ECM1 upregulation (Figure 2A). Consistently, treatment with 5 µg/ml 5-FU induced significant time-dependent increases in ECM1 protein level expression relative to control treatment (Figure 2B). Subsequent qRT-PCR and Western immunoblotting-based comparison revealed higher levels of ECM1 expression in HCT15/FU cells relative to parental cells (Figures 2C,D).

Following that, cell lines with knocked-down and overexpressed ECM1 were generated, with qRT-PCR and Western immunoblotting utilized to confirm transfection effectiveness (Figures 2E–H). Analysis of cell proliferation and sensitivity to 5-FU using the cell counting kit-8 (CCK-8) and colony forming assay showed that ECM1 knockdown reduced HCT15/FU cell resistance to 5-FU (Figures 2I,J). In contrast, the overexpression of ECM1 conversely rendered HCT15 cells more resistant to this chemotherapeutic drug (Figures 2K,L). These data suggest that ECM1 is closely linked to 5-FU resistance.

### 5-Fluorouracil-resistant colorectal cancer cells exhibit epithelial-mesenchymal transition induction

**FIGURE 3:**
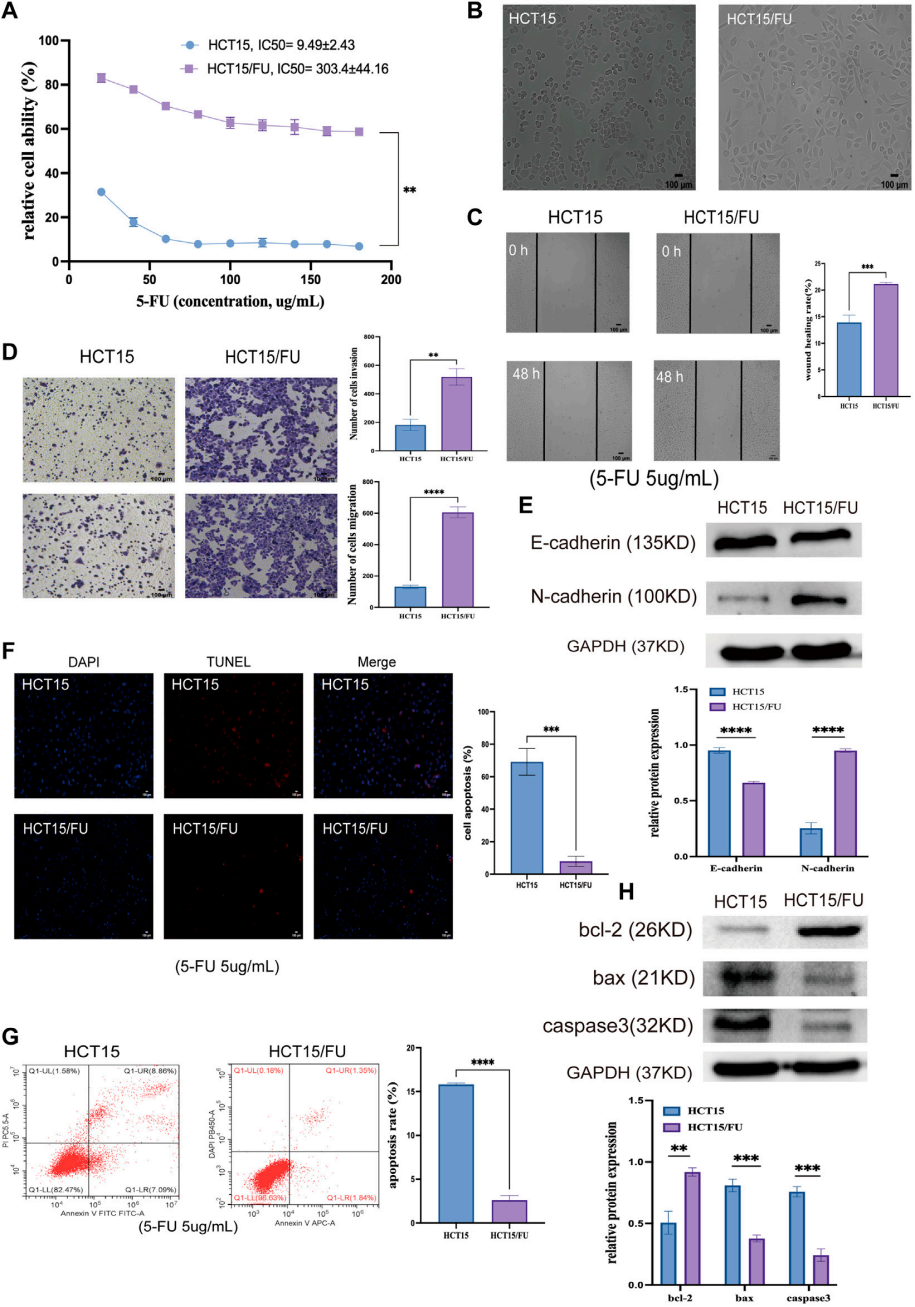
5-FU-resistant CRC cells exhibit EMT induction and apoptosis-resistant phenotypes. **(A)** CCK-8 assays were used to compute IC50 and resistance index (RI) values for HCT15 and HCT15/FU cells. **(B)** HCT15 and HCT15/FU cell morphological characteristics were visualized. **(C,D)** Transwell and wound healing experiments investigated these cells’ relative migratory and invasive activity. **(E)** Both HCT15 and HCT15/FU cells have EMT-related protein levels. **(F,G)** TUNEL staining and flow cytometry were employed to examine the apoptotic death of HCT15 and HCT15/FU cells. **(H)** Apoptosis-associated protein expression was detected in HCT15/FU cells. Data are represented as means ± SD (**p < 0.01, ***p < 0.001, ****p < 0.0001).

HCT15 and HCT15/FU cells were exploited to examine the molecular basis for this chemoresistant trait. Initial CCK-8 experiments validated the resistance of HCT15/FU cells to 5-FU as indicated by respective 5-FU IC50 values for HCT15 and HCT15/FU cells of 9.49 ± 2.43 µg/ml and 303.4 ± 44.16 µg/ml, with a computed resistance index (RI) of 33.83 ± 11.53 (Figure 3A). These HCT15/FU cells also exhibited morphological characteristics distinct from those of parental HCT15 cells including spindle-shaped characteristics and fewer contacts among cells (Figure 3B), suggesting that these resistant cells had undergone the EMT process. To verify this hypothesis, wound healing and transwell experiments were performed to assess the relative migratory and invasive capacity of HCT15 and HCT15/FU cells, revealing that HCT15/FU cells were much more robust in these activities (Figures 3C,D). Western immunoblotting observed higher mesenchymal N-cadherin levels and lower epithelial E-cadherin expression levels in HCT15/FU cells (Figure 3E). Our findings showed that EMT induction could contribute to 5-FU-resistant CRC cell growth.

### 5-Fluorouracil-resistant colorectal cancer cells are resistant to apoptosis induction

Next, TUNEL staining was used to analyze the apoptosis of HCT15 and HCT15/FU cells upon treatment for 48 h with 5-FU (5 µg/ml). Significantly lower apoptosis rates were observed for HCT15/FU cells under these conditions than for HCT15 cells (Figures 3F,G). These HCT15/FU cells consistently exhibited enhanced anti-apoptotic Bcl-2 protein levels and reduced pro-apoptotic Bax and caspase-3 levels under these conditions, as determined *via* Western immunoblotting (Figure 3H). Consequently, HCT15/FU cells may have an inherent resistance to apoptosis, which may explain why they are resistant to 5-FU.

### Extracellular matrix 1 regulates epithelial-mesenchymal transition phenotypes

**FIGURE 4:**
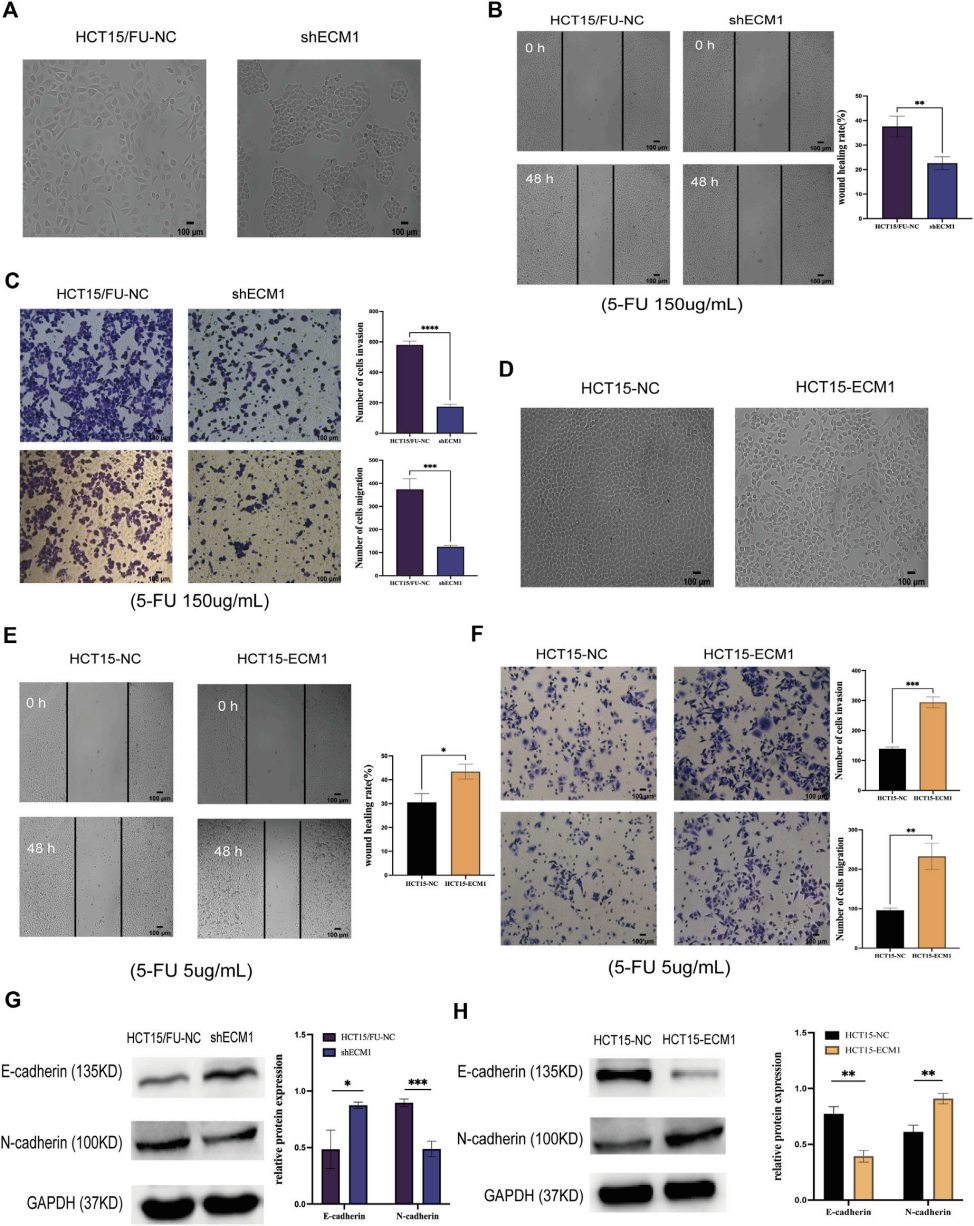
Knocking down ECM1 alters CRC cell EMT induction. The morphology of the respective HCT15 cell types was examined when ECM1 was either knocked down **(A)** or overexpressed **(D)**. **(B,C)** A high dose of 5-FU (150 µg/ml) was used to treat HCT15/FU-NC and shECM1 cells, after which their migration/invasion was assessed through wound healing and Transwell assays. **(E,F)** A 5 µg/ml dose of 5-FU was used to treat HCT15-NC and HCT15-ECM1 cells, after which their migration/invasion was detected through Transwell and wound healing assays. **(G,H)** EMT-associated protein expression was detected following ECM1 knocked down or overexpressed. Data are means ± SD (*p < 0.5, **p < 0.01, ***p < 0.001, ****p < 0.0001).

ECM1 knockdown contributed to morphological changes in HCT15/FU cells (Figure 4A), with a corresponding drop in invasion and migration (Figures 4B,C). Knocking down ECM1 contributed to a significant decrease in N-cadherin protein levels in these cells and a concomitant increase in E-cadherin levels (Figure 4G). Notably, the opposite phenotypic changes were observed when ECM1 was overexpressed in HCT15 parental cells (Figures 4D–F,H). These results strongly suggested that ECM1 can modulate 5-FU sensitivity in CRC cells *via* regulating EMT induction.

### Extracellular matrix 1 expression alters the resistance of 5-Fluorouracil-treated colorectal cancer cells to apoptotic death

**FIGURE 5:**
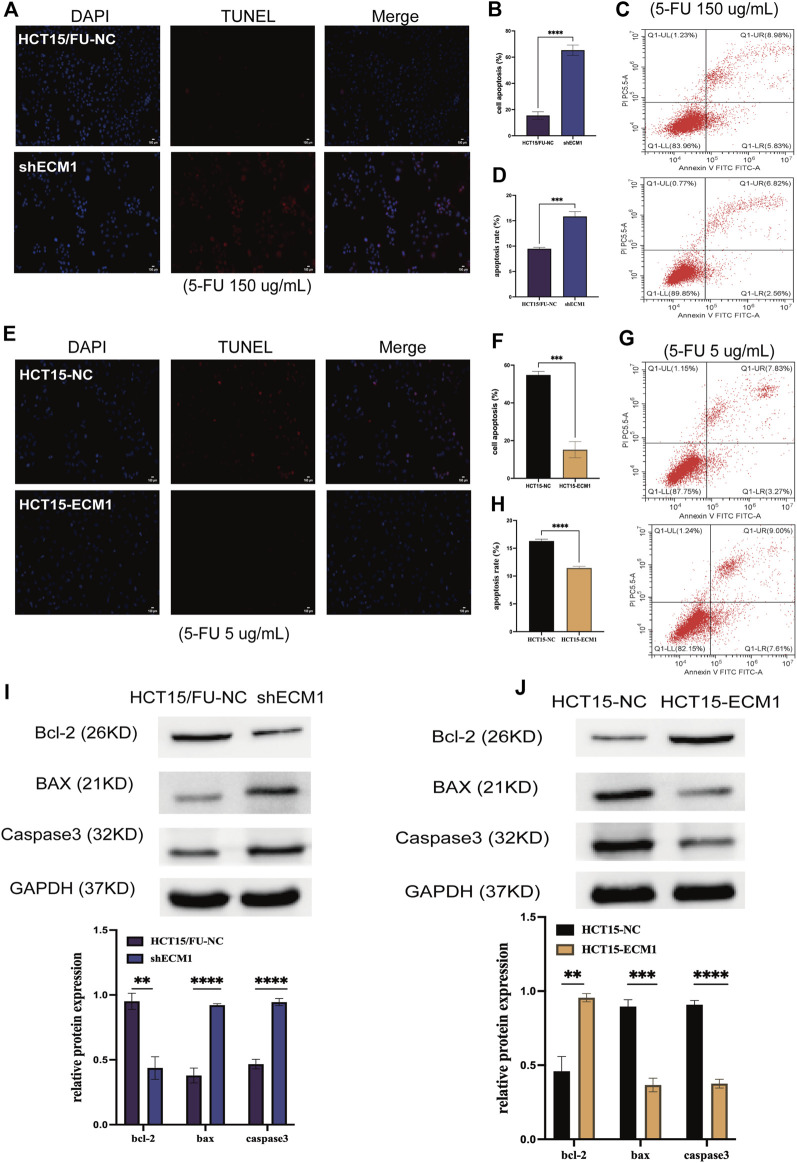
ECM1 regulates cellular resistance to 5-FU-induced apoptosis. The apoptotic death of 5-FU-treated cells was examined through TUNEL staining and flow cytometry. **(A–H)** The impact of altered ECM1 expression on the apoptosis of 5-FU-treated cells. **(I,J)** Apoptosis-related protein expression was detected *via* Western immunoblotting. Data are means ± SD (*p < 0.5, **p < 0.01, ***p < 0.001, ****p < 0.0001).

Next, apoptosis was evaluated in HCT15/FU cells treated with a high dosage of 5-FU (150 µg/ml) for 24 h using TUNEL labeling and flow cytometry after the expression of ECM1 was knocked down or not. ECM1 knockdown significantly increased apoptotic death in these cells using this method (Figures 5A–D). HCT15/FU cells with ECM1 knockdown consistently showed increased Bax and caspase-3. They decreased Bcl-2 levels in Western immunoblotting (Figure 5I). The opposite phenotypes were observed following the overexpression of ECM1 in HCT15 cells treated with 5-FU (5 µg/ml) (Figures 5E–H,J). Consequently, our findings supported the hypothesis that ECM1 regulates CRC cell resistance to 5-FU-induced apoptosis.

### Extracellular matrix 1 controls colorectal cancer cell resistance to 5-Fluorouracil by regulating the PI3K/AKT/GSK3β pathway

**FIGURE 6:**
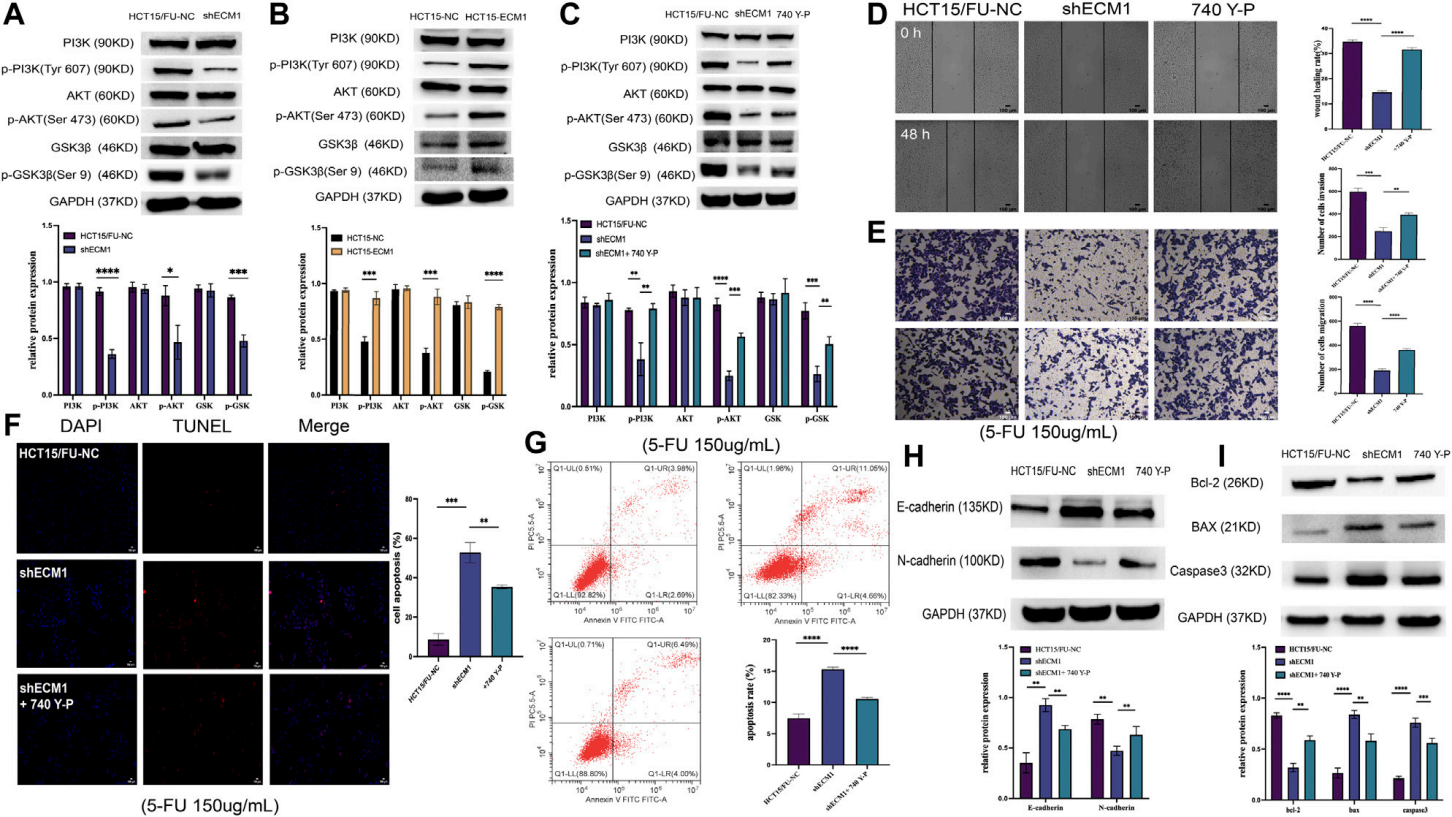
ECM1 enhances CRC cell resistance to 5-FU *via* the PI3K/AKT/GSK3β pathway. **(A,B)** The indicated PI3K/AKT pathway proteins and phosphoproteins were detected in HCT15/FU or HCT15 cells in which ECM1 was silenced or overexpressed. **(C)** PI3K/AKT-related protein expression was detected following 740 Y-P treatment for 24 h to confirm treatment efficacy. **(D–G)** After treatment with 740 Y-P for 24 h, the invasive and migratory activities of HCT15/FU-shECM1 cells were examined *via* Transwell and wound healing analyses **(D,E)**. In contrast, their apoptotic death was detected *via* TUNEL staining and flow cytometric analyses **(F,G)**. **(H,I)** Western immunoblotting was used to identify proteins involved in apoptosis and EMT. The data are shown as means ± SD (*p < 0.05, **p < 0.01, ***p < 0.001, ****p < 0.0001).

It has been shown that PI3K/AKT/GSK3β signaling is crucial for the development of chemoresistance in tumor cells (Shi et al., 2022; Zhang D et al., 2022). As a result, the next step was to investigate how this PI3K/AKT/GSK3β pathway is regulated by ECM1 to control 5-FU resistance in HCT15 cells. ECM1 knockdown decreased the phosphorylation of PI3K, AKT, and GSKβ3 without having a substantial impact on the overall levels of these proteins in HCT15/FU cells, according to the Western blotting analysis of the protein parts of this pathway (Figure 6A). Conversely, increased p-PI3K, p-AKT, and p-GSK3β levels were evident in HCT15 cells upon ECM1 overexpression (Figure 6B).

The PI3K agonist 740 Y-P was subsequently used to treat HCT15/FU cells in which ECM1 had been silenced to formally test the relationship between ECM1-mediated chemoresistance and PI3K/AKT/GSK3β signaling. Protein levels associated with this signaling were detected using Western immunoblotting. Our data showed that 740 Y-P could restore the PI3K, AKT, and GSK3β phosphorylation in ECM1 knockdown HCT15/FU cells (Figure 6C). Moreover, 740 Y-P administration was shown to improve the capacity of these cells to move dramatically, invade, avoid apoptotic death (Figures 6D–G) and the resistance to 5-FU (Supplementary Figure S1A), effectively overcoming the adverse impact of ECM1 knockdown. According to these findings, the 740 Y-P treatment increased the levels of Bcl-2 and N-cadherin in these cells while reducing the production of Bax, caspase-3, and E-cadherin (Figures 6H,I). The opposite effects were observed upon the treatment of HCT15 cells in which ECM1 was overexpressed with LY294002, inhibiting PI3K/AKT pathway signaling (Supplementary Figures S1B, S2). Overall, these findings imply that ECM1 regulates the PI3K/AKT/GSK3 pathway, at least in part, to alter CRC cells’ sensitivity to 5-FU.

### Extracellular matrix 1 regulates *in vivo* colorectal cancer tumor resistance to 5-Fluorouracil *via* the PI3K/AKT/GSK3β pathway-mediated control of epithelial-mesenchymal transition induction and apoptosis

**FIGURE 7:**
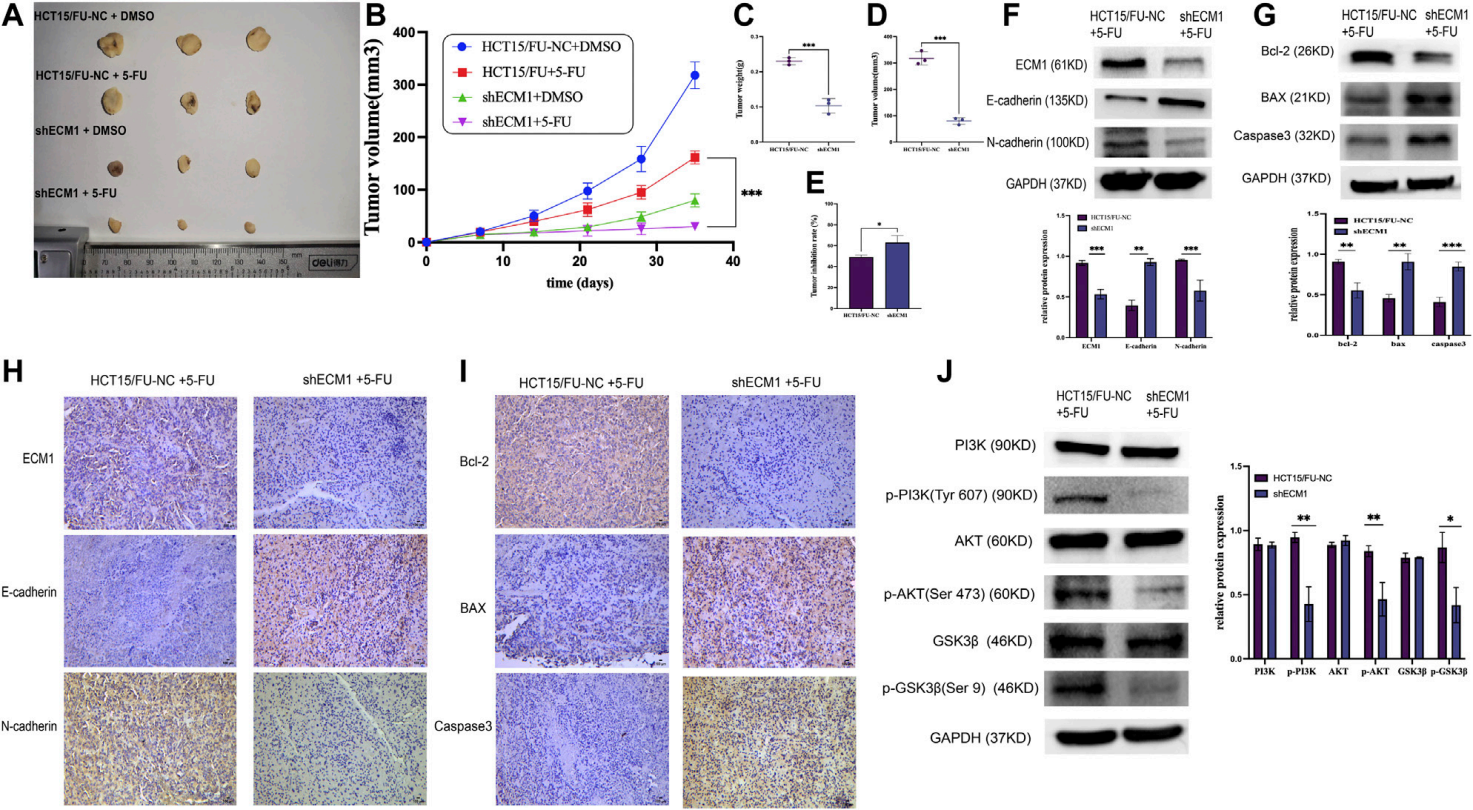
Knocking down ECM1 enhances the *in vivo* chemosensitivity of HCT15/FU cells. **(A–E)** Knocking down ECM1 enhanced the 5-FU sensitivity of tumor cells, as evidenced by decreased tumor volume and weight on day 28 post-treatment with 5-FU. **(F–I)** Western immunoblotting and IHC staining were used to detect apoptosis-, EMT- and pathway-related proteins. Data are means ± SD (*p < 0.05, **p < 0.01, ***p < 0.001). **(J)** Western immunoblotting was used to detect PI3K/AKT-related protein expression.

A mouse xenograft model system was then created to extend the abovementioned results *in vivo*. Mice were randomly allocated to the following groups (*n* = 3/group): HCT15/FU-NC (control), HCT15/FU-NC (5-FU treatment), HCT15/FU-shECM1 (control), and HCT15/FU-shECM1 (5-FU treatment). Monitoring these mice revealed that the knockdown of ECM1 significantly reduced tumor weight and volume (Figures 7A–D). 5-FU efficacy in these animals was also detected by measuring inhibition rate values upon 5-FU treatment, revealing a significantly higher inhibition rate in the HCT15/FU-shECM1 group relative to the HCT15/FU-NC group upon treatment with this chemotherapeutic drug (Figure 7E). Western blotting and IHC consistently exhibited increased intratumoral Bax, caspase-3, and E-cadherin levels and reduced N-cadherin and Bcl-2 levels of HCT15/FU-shECM1 (5-FU treatment) compared with HCT15/FU-NC (5-FU treatment) (Figures 7F–I). Meanwhile, knockdown of ECM1 significantly reduced the PI3K, AKT, and GSK3β phosphorylation in HCT15/FU cells *in vivo* (Figure 7J). These results demonstrated that knocking down ECM1 was sufficient to restore 5-FU sensitivity in 5-FU resistant CRC cells *in vivo via* altering EMT induction and apoptotic resistance *via* the PI3K/AKT/GSK3β pathway.

## Discussion

For the treatment of CRC, 5-FU continues to be among the most popular and effective chemotherapeutic medicines (Glimelius et al., 2021), and the adjuvant therapy based on 5-FU is essential to CRC patients in stage II-IV after radical surgery, which could prolong the survival time of CRC patients (Wilkinson et al., 2010; Miller et al., 2019; Huang X et al., 2022). The emergence of chemoresistance remains a significant barrier to long-term benefit (Xie et al., 2020). Therefore, effective strategies are needed to solve this problem.

ECM1 has been proved to play an essential role in embryonic chondrogenesis (Deckers et al., 2001), skin differentiation (Mcgrath, 2015), epithelial cell proliferation (Yang et al., 2020), Angiogenesis (Wang B et al., 2020), tumorigenesis (Wang et al., 2003) and other biological processes. Emerging evidence has revealed that ECM1 is upregulated in many cancers and contributes to a worse patient prognosis (Lee et al., 2015; Chen et al., 2016; Gan et al., 2018). Previously, ECM1 was found to influence various epithelial malignancies’ progression through the modulation of cellular proliferative activity (Wang et al., 2019), tumor metastasis (Wu et al., 2019) and drug resistance (Lee et al., 2014; Lee and Lee, 2022; Lv et al., 2022). For instance, ECM1 activated the epidermal growth factor receptor (EGFR) signaling pathway and facilitated the progression of breast cancer cells and their resistance to trastuzumab treatment (Lee et al., 2014). ECM1 also regulated the acquired endocrine resistance in ER breast cancers by activating Src (Lee and Lee, 2022). In ovarian cancer, ECM1 was associated with cisplatin resistance, and high levels of ECM1 in the stroma can induce normal fibroblasts transformation and then promote tumor progression (Lv et al., 2022). This study aims to determine ECM1’s contribution to CRC treatment resistance. First, we assembled a group of CRC patients who underwent radical surgery and 5-FU treatment. The findings demonstrated that reduced 5-FU sensitivity and worse OS and DFS were related to ECM1 overexpression. In addition, we found that the treatment of 5-FU could induce the expression of ECM1 of HCT15, and ECM1 expression was significantly upregulated in HCT15/FU cells at both mRNA and protein levels compared with HCT15. Furthermore, we conducted colony formation and CCK-8 assays, which revealed ECM1 knockdown to increase the 5-FU sensitivity of HCT15/FU cells. These results indicated that the ECM1 expression level is tightly related to CRC resistance to 5-FU.

Drug resistance is one major cause of chemotherapy failure and cancer mortality (Chern and Tai, 2020; Sun et al., 2020), and verifying the underlying molecular mechanism is urgently needed. Drug resistance is often accompanied by increased cellular growth pathways and enhanced metastatic potential (Dongre and Weinberg, 2019). Epithelial-mesenchymal transition (EMT) is one of the leading mechanisms of tumor invasion and metastasis (Han et al., 2022; Huang Y et al., 2022). Following EMT induction, epithelial cells acquire a mesenchymal-like phenotype (Shibue and Weinberg, 2017), providing a level of plasticity related to the ability of cells to respond to treatment, with mesenchymal cells being better able to resist an array of treatments (Yuan et al., 2020; Luo et al., 2021). Previous research has shown that EMT and cell apoptosis may participate in the process of 5-FU resistance (Li et al., 2018; Shi et al., 2020; Wang J et al., 2020; Xie et al., 2021). In our study, the HCT15/FU cells were consistently found to exhibit mesenchymal characteristics, including spindle-shaped morphology and enhanced migratory and invasive activity coinciding with increased N-cadherin expression and reduced E-cadherin levels. Additionally, HCT15/FU cells demonstrated strong resistance to 5-FU-induced apoptosis, accompanied by changes in the levels of associated proteins.

In our previous study, we found that ECM1 could promote CRC progression by influencing cell growth, metastasis and the process of EMT (Long et al., 2022). As a result, we postulated that cell apoptotic evasion and EMT might be regulated by ECM1 in the HCT15/FU cells with mesenchymal-like phenotypes. TUNEL, flow cytometry, Transwell, and wound healing experiments demonstrated that ECM1 downregulation could reverse EMT while increasing 5-FU-induced cell apoptosis. When EMT-related and apoptotic proteins were further evaluated *via* Western blotting, ECM1 knockdown was found to enhance the expression of E-cadherin, Bax and caspase 3 but decrease N-cadherin and BCL-2, respectively. On the other hand, when ECM1 was overexpressed, the opposite result was seen. These findings demonstrated that ECM1-induced EMT and cell death might be responsible for CRC cells’ resistance to 5-FU.

PI3K/AKT/GSK3β signaling activity is an essential regulator of diverse malignant behaviour in tumor cells (Hu et al., 2022; Wang et al., 2022), including apoptotic induction (Zhang Y et al., 2022), EMT induction (Zhao et al., 2019; Jiang et al., 2020), and resistance to treatment (Kashyap et al., 2018). Previous studies showed that the combination of PI3K/AKT inhibitor LY294002 and 5-FU could lead to higher cytotoxic efficacy by inducing a greater extent of cell apoptosis than single drug therapy (Feng et al., 2018; Ni et al., 2022). Additionally, AKT/GSK3β signaling was found to be activated in CRC-resistant cells and controlled HIF1α expression (Shi et al., 2022). Moreover, ECM1 has been shown to regulate PI3K/AKT/GSK3β/Snail signaling in CRC (Long et al., 2022). In line with these investigations, we found that ECM1-regulated 5-FU resistance in CRC was mediated through PI3K/AKT/GSK3β. The outcomes demonstrated that knocking down ECM1 reduces the phosphorylation of PI3K, GSK3β, and AKT without changing the level of total proteins. The PI3K/AKT agonist 740 Y-P significantly suppressed apoptotic death, increased invasion, migration and the resistance to 5-FU in ECM1-knocked down HCT15/FU cells, all while downregulating Bax, caspase-3, and E-cadherin expression and upregulating Bcl-2 and N-cadherin. The opposite was observed following LY294002-mediated PI3K/AKT inhibition in HCT15 cells overexpressing ECM1. Murine xenograft models further demonstrated that silencing ECM1 within HCT15/FU cells enhanced their *in vivo* sensitivity to 5-FU, with corresponding reductions in N-cadherin, Bcl-2, p-AKT, p-PI3K, and p-GSK3β expression and increased Bax, caspase-3 levels, and E-cadherin, suggesting the ability of ECM1 to regulate the PI3K/AKT/GSK3β pathway to control CRC cell EMT induction and apoptotic resistance *in vitro* and *in vivo*. These results suggest that ECM1 modulates cell apoptosis and EMT to modify the sensitivity of CRC cells to 5-FU *via* PI3K/AKT/GSK3β pathway. Of course, there are still some limitations of our study. For instance, the precise molecular mechanism by which ECM1 induces phosphorylation of the key molecules of PI3K/AKT/GSK3β pathway remains unclear. Therefore, future research will focus on the molecular processes behind ECM1-mediated PI3K/AKT activation during CRC resistance to 5-FU.

## Conclusion

In conclusion, our current study showed that patients resistant to 5-FU exhibited higher levels of ECM1 expression, and patients with high expression of ECM1 tend to have a shorter DFS and OS. Moreover, ECM1-induced EMT and evasion of cell apoptosis caused the process of CRC cells resisting to 5-FU, which was regulated by PI3K/AKT/GSK3β signaling pathway. Accordingly, these data highlight ECM1 as a promising therapeutic target for efforts to overcome CRC resistance to 5-FU treatment.

## Data Availability

The raw data supporting the conclusion of this article will be made available by the authors, without undue reservation.

## Supplementary material

SUPPLEMENTARY FIGURE S1 ECM1 enhances CRC cell resistance to 5-FU *via* the PI3K/AKT/GSK3β pathway. After being treated with PI3K agonist (740 Y-P) or PI3K inhibitor (LY294002) for 24 h, the CCK8 assay was used to detect the change in drug sensitivity. **(A)** the effects of 740 Y-P on 5-FU resistance in HCT15/FU cells. **(B)** the effect of PI3K LY294002 on 5-FU sensitivity of HCT15 cells.

SUPPLEMENTARY FIGURE S2 Inhibiting PI3K/AKT pathway activation reversed the harmful effects of ECM1 overexpression in 5-FU-treated HCT15 cells. **(A,B)** PI3K/AKT-related protein expression was detected following LY294002 treatment for 24 h to confirm treatment efficacy. **(C–G)** Following LY294002 treatment, the migratory and invasive activity levels of HCT1-ECM1 cells were examined through wound healing, Transwell assays, TUNEL labeling and flow cytometric studies revealed that these cells had undergone apoptosis **(H–K)** Western immunoblotting was used to identify proteins involved in EMT and apoptosis. The data are shown as means ± SD (**p* < 0.05, ***p* < 0.01, ****p* < 0.001, *****p* <0.0001).
