# Supplementary figures and images for: ECM1 regulates the resistance of colorectal cancer to 5-FU treatment by modulating apoptotic cell death and epithelial-mesenchymal transition induction
Source: Front Pharmacol. 2022 Nov 2;13:1005915. doi: 10.3389/fphar.2022.1005915 (PMC9666402; doi:10.3389/fphar.2022.1005915)

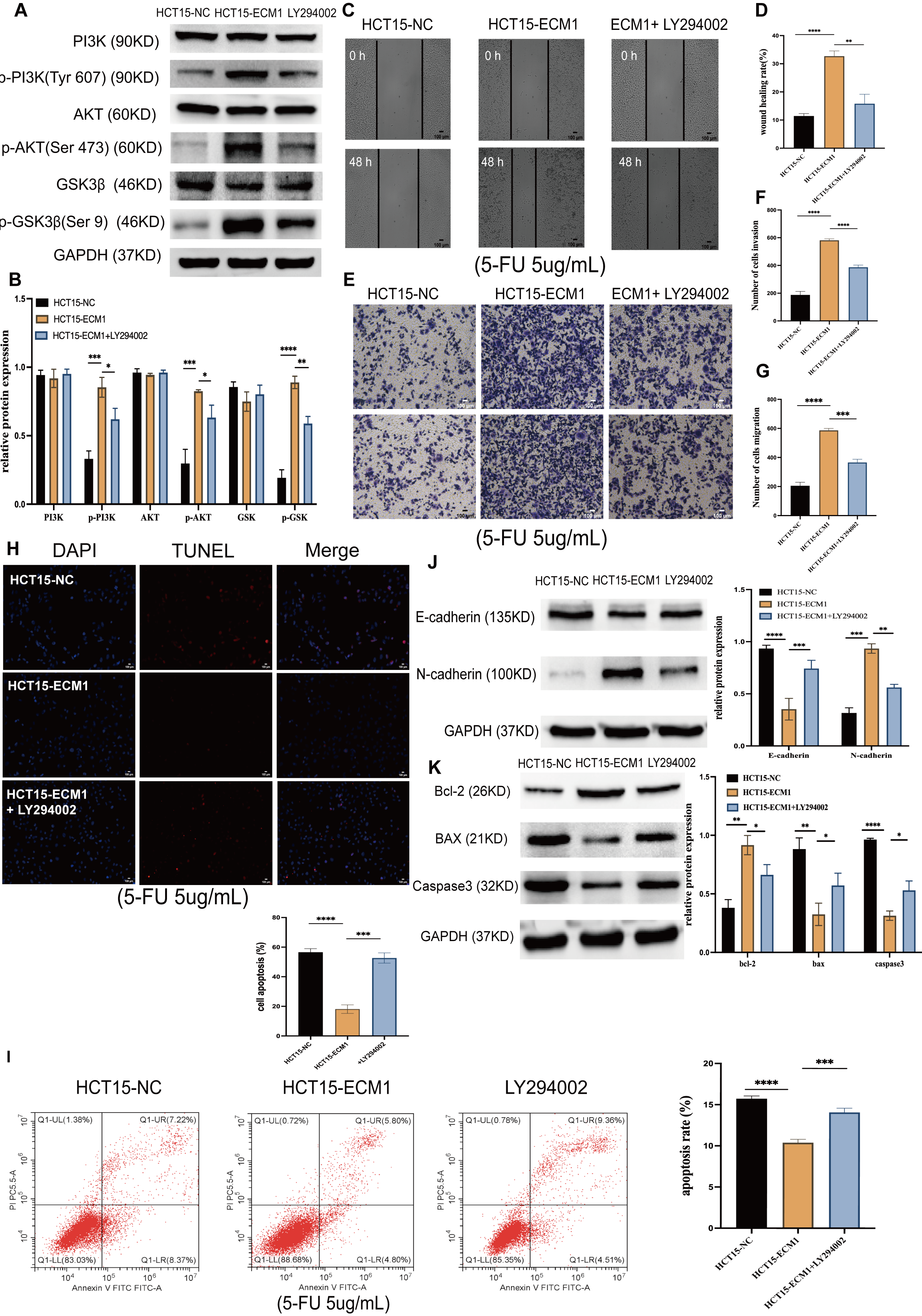

Supplement: Supplementary file 1 [file Image2.TIF]

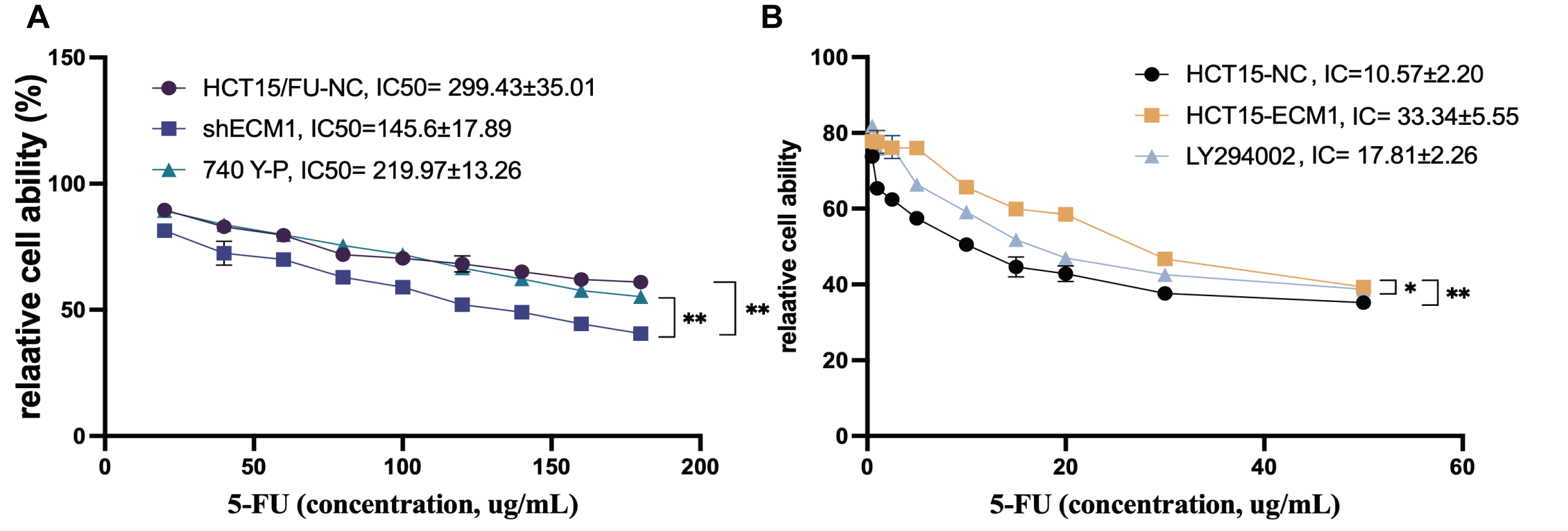

Supplement: Supplementary file 2 [file Image1.TIF]
